# Barley responses to combined waterlogging and salinity stress: separating effects of oxygen deprivation and elemental toxicity

**DOI:** 10.3389/fpls.2013.00313

**Published:** 2013-08-14

**Authors:** Fanrong Zeng, Lana Shabala, Meixue Zhou, Guoping Zhang, Sergey Shabala

**Affiliations:** ^1^School of Agricultural Science and Tasmanian Institute of Agriculture, University of TasmaniaHobart, TAS, Australia; ^2^Department of Agronomy, College of Agriculture and Biotechnology, Zhejiang UniversityHangzhou, China

**Keywords:** salinity, waterlogging, microelement toxicity, barley, breeding, manganese, potassium, sodium

## Abstract

Salinity and waterlogging are two major factors affecting crop production around the world and often occur together (e.g., salt brought to the surface by rising water tables). While the physiological and molecular mechanisms of plant responses to each of these environmental constraints are studied in detail, the mechanisms underlying plant tolerance to their combined stress are much less understood. In this study, whole-plant physiological responses to individual/combined salinity and waterlogging stresses were studied using two barley varieties grown in either vermiculite (semi-hydroponics) or sandy loam. Two weeks of combined salinity and waterlogging treatment significantly decreased plant biomass, chlorophyll content, maximal quantum efficiency of PSII and water content (WC) in both varieties, while the percentage of chlorotic and necrotic leaves and leaf sap osmolality increased. The adverse effects of the combined stresses were much stronger in the waterlogging-sensitive variety Naso Nijo. Compared with salinity stress alone, the combined stress resulted in a 2-fold increase in leaf Na^+^, but a 40% decrease in leaf K^+^ content. Importantly, the effects of the combined stress were more pronounced in sandy loam compared with vermiculite and correlated with changes in the soil redox potential and accumulation of Mn and Fe in the waterlogged soils. It is concluded that hypoxia alone is not a major factor determining differential plant growth under adverse stress conditions, and that elemental toxicities resulting from changes in soil redox potential have a major impact on genotypic differences in plant physiological and agronomical responses. These results are further discussed in the context of plant breeding for waterlogging stress tolerance.

## Introduction

Excessive soil salinization is a major ecological and agronomic problem throughout the world. According to the FAO Land and Plant Nutrition Management Service (2008), more than 800 Mha of land throughout the world is affected by salinity, which accounts for over 6% of the world's total land area. Apart from natural salinity, secondary (human-induced) salinization of arable land has become a serious threat to agricultural production owing to improper cultivation practices and irrigation (Pannell and Ewing, 2004). Excessive quantities of ions (mainly Na^+^ and Cl^−^) in the soil solution decrease soil osmotic potentials and the availability of water to plant roots. When accumulated in the shoot, these ions induce ion toxicity by disrupting the structure of enzymes, damaging cell organelles and interfering with cell metabolism (Maathuis and Amtmann, 1999). Salinity stress also results in significant ROS accumulation, both in roots (Xie et al., 2011) and leaves (Tanou et al., 2009), and interferes with K^+^ homeostasis (Maathuis and Amtmann, 1999; Shabala and Cuin, 2008), triggering accelerated cell death (Shabala, 2009; Joseph and Jini, 2010).

Waterlogging occurs over a vast region of the world, adversely affecting about 10% of the global land area (Setter and Waters, 2003) and reducing crop yields by as much as 80% (Shabala, 2011). Under waterlogging condition, soil gas exchange is severely impeded. This results in a significant depletion of free oxygen (O_2_) and accumulation of carbon oxide (CO_2_) due to microbial and root respiration (Bailey-Serres and Voesenek, 2008). As soon as the free O_2_ surrounding the roots is depleted, hypoxia stress occurs, causing a transfer from aerobic to anaerobic metabolism in roots, with dramatic restrictions to ATP synthesis (Barrett-Lennard, 2003; Teakle et al., 2006). Waterlogging also causes a sharp decrease in the soil redox potential, resulting in very significant changes to the soil chemical profile. Effects include a changed availability of mineral substances, reduction of manganese (Mn^4+^), iron (Fe^3+^), and sulfate (SO^2−^_4_), increased solubility of potentially toxic metals and production of toxic compounds by plant roots and microbial anaerobic metabolism (Kozlowski, 1997; Shabala, 2011). As a consequence of these changes, plants show altered membrane transport, decreased stomatal conductance and leaf water potentials, enhanced root senescence, reduced root and shoot growth, and eventually, death of the whole plant (Barrett-Lennard, 2003).

In recent years, an impressive amount of knowledge has accumulated on plant physiological and molecular responses to salinity or waterlogging stresses. However, studies dealing with the combined effects of these two stresses are much rarer and often controversial [reviewed by Barrett-Lennard (2003)]. Nonetheless, the occurrence of combined salinity and waterlogging stress is increasing throughout the world This is due to intensive irrigation in agricultural production systems (Smedema and Shiati, 2002), rise of saline water tables (Hatton et al., 2003), and seawater intrusion in coastal environments (Carter et al., 2006). When combined with waterlogging, salinity can cause even greater damage to plants, so having a major impact on agricultural production (Barrett-Lennard, 2003). Only a very few crop species can tolerate the combination of salinity and waterlogging (Bennett et al., 2009), and the physiological and molecular mechanisms conferring this tolerance remain elusive.

Barley (*Hordeum vulgare* L.) is one of the most important crop species in the world. Barley is tolerant to salinity (Chen et al., 2007), so is a good candidate for use in saline discharge areas. However, barley is sensitive to waterlogging (Zhou et al., 2012), and the co-occurrence of waterlogging with salinity may seriously decrease this potential. In this study, the physiological and ionic characteristics of two contrasting barley varieties, grown in two soil types, were investigated in response to combined salinity and waterlogging stress. Our results suggest that hypoxia is not the only factor affecting plant growth under adverse stress conditions and that elemental toxicities resulting from changes in the soil redox potential has a major impact on genotypic differences in plant physiological and agronomical responses.

## Materials and methods

### Plant materials and growth conditions

Two barley (*Hordeum vulgare* L.) varieties contrasting in both waterlogging and salinity tolerance (CM72, tolerant to salinity and with medium tolerance to waterlogging, and Naso Nijo, sensitive to both waterlogging and salinity; Chen et al., 2007; Pang et al., 2007), were used in this work. Seeds were obtained from the Australian Winter Cereal Collection and multiplied using the TIA facilities in Launceston. Seeds were surface sterilized with 10% commercial bleach (NaClO 42 g/L, Pental Products, Shepparton, Australia), thoroughly rinsed with tap water, then sown at a 10 mm-depth in 2 L-pots. Two different types of growth media—sandy loam and vermiculite—were used. Sandy loam soil, which was taken from the University of Tasmania farm near Cambridge in southeast Tasmania, was first air-dried and then sieved through a 5 mm sieve. The soil was fertilized by adding all the essential macronutrients at the optimal field application rates, taking into account the pot factor (in g/10 L: 5.13 NH_4_NO_3_, 13.57 NaH_2_PO_4_, 4.88 K_2_SO_4_, 3.1 CaSO_4_, and 1.42 MgCl_2_) and watered to field capacity with tap water during the experiment. Plants grown in vermiculite were watered with half-strength Hoagland's nutrient solution. After germination, barley seedlings were thinned to 10 uniform and healthy plants in each pot. Plants were grown under controlled glasshouse condition (with a day-length of 14 h; light/dark temperatures, 25/15°C; and relative humidity, 65%) at the University of Tasmania (Hobart, Australia). The experiment was repeated twice in April and June 2012.

Treatments were imposed when plants were at the fully expanded first leaf stage (~1 week old). For treatments with waterlogging, pots were placed into large black tanks (6 pots in each tank). Four treatments were given: control (no NaCl; well drained), salinity (referred as “NaCl”; well drained pots watered with 200 mM NaCl solution), waterlogging (“WL”; submerged pots; no NaCl) and combined waterlogging and salinity (“NaCl/WL”; pots submerged in 200 mM NaCl solution). Waterlogged conditions were created by using either tap water or half-strength Hoagland's nutrient solution, depending on the soil type (i.e., whether sandy loam or vermiculate). The entire experiment was carried out in a split-plot design with tanks as the main plots and barley varieties as subplots. Six replicates were set up for each treatment × variety combination.

Daily irrigation for drained treatments was artificially maintained with the same amount (150 mL) of different solutions, as described above. The water level of waterlogged treatments was kept 15 mm above the soil surface. Plants were subjected to different treatments for 14 days, after which the genotypic variance was clearly distinguished visually. The seedlings were then sampled randomly for the following analyses.

### SPAD and chlorophyll fluorescence

Leaf chlorophyll content and chlorophyll fluorescence were measured on the middle part of the oldest fully expanded leaves (which had been subjected to the treatment for a full 2 weeks), prior to sampling for other parameters. Leaf chlorophyll content was measured with a SPAD meter (SPAD-502, MINOLTA, Japan). Chlorophyll fluorescence was measured with a OS-30p chlorophyll fluorometer (OPTI-Sciences, Hudson, USA). Plants were dark-adapted for 30 min prior to measurement. The maximum quantum efficiency of photosystem II (*F*v/*F*m = (*F*m–*F*o)/*F*m) was recorded at a saturating actinic light (660 nm) intensity of 1100 μmol/m^2^/s. Twelve replicates were randomly taken for each treatment × variety combination.

### Ratio of chlorotic and necrotic leaves

Prior to harvest for biomass, the number (no.) of chlorotic, necrotic, and total leaves from each plant were counted. The ratio of chlorotic (or necrotic) leaves was then calculated according to the equation: ratio of chlorotic (or necrotic) leaves = no. of chlorotic (/necrotic) leaves/no. of total leaves. Twelve replicates were randomly taken for each treatment × variety combination.

### Biomass

For harvesting, plant roots were gently washed with running tap water, rinsed with distilled water, then blotted dry with soft tissue. Five replicates were taken for each treatment × variety combination, and three plants were bulked together for each replicate. The fresh weights of shoots and roots were measured as soon as the seedlings were separated and the dry weights were measured after drying in a Unitherm Drier (Birmingham, England) for 2 days at 65°C.

### Leaf water content

Leaf fresh and dry weights were used to calculate the leaf water content (WC) on a fresh weight basis using the following equation: WC% = (fresh weight - dry weight)/fresh weight × 100%. Fresh leaf blades of whole seedlings were collected and weighed immediately for their fresh weights. Dry weights were determined after drying for 2 days at 65°C. Five replicates were taken for each treatment × variety combination.

### Na^+^ and K^+^ contents and osmolality in plant tissue

Na^+^ and K^+^ contents were determined from the leaf and root sap. After harvesting as described above, plant roots and the oldest fully expanded leaves, which had been subjected to the treatment for a full 2 weeks, were collected and immediately stored in a 1.5 ml microcentrifuge tube at −20^°^C. Five replicates were taken for each treatment × variety combination. The sap from roots and leaves was extracted by the freeze-thaw method (Cuin et al., 2008). After centrifuged at 10,000 g for 3 min, the extracted sap sample was diluted 500 times with double distilled water and analyzed for its Na and K content using a flame photometer (PF97, VWR International, Murarrie, Australia).

The extracted leaf sap was also analyzed for its osmolality using a vapor pressure osmometer (Vapro, Wescor Inc. Logan, Utah, USA).

### Root ATP content

Two weeks after commencing treatments, roots were harvested for ATP extraction according to a modified method of Yang et al. (2002). Briefly, 0.2 g of liquid nitrogen-homogenized root powder was rapidly mixed with 0.5 mL 0.0005% (v/v) HCl.O_4_ and heated in a boiling water bath for 10 min. After cooling on ice, the extraction mixture was centrifuged for 5 min at 10,000 × g at 4°C. The supernatant was used for the ATP assay. The ATP content was quantified using the ATP Colorimetric Assay Kit (ab83355, ABCAM, Cambridge, UK) with a microplate reader (SPECTROstar Nano, BMG LABTECH, Mornington, Australia). The reaction mix contained 4% (v/v) ATP probe, 4% (v/v) ATP converter and 4% (v/v) developer in the ATP Assay Buffer (ab83355, ABCAM, Cambridge, UK). ATP standards (0–5 nmol range) and extracted samples were placed into a 96-well plate. The reaction mix was added at 1:1 (v/v) ratio, and plates were incubated at the room temperature in the dark for 30 min. The extraction solution without root tissue was used as a negative control. ATP concentrations in the samples were calculated by plotting the measured absorbance at 570 nm (OD_570 nm_) vs. the standard linear curve.

### Net ion fluxes from the root epidermis

Net K^+^ and H^+^ fluxes were measured from the root epidermis of barley seedlings using non-invasive ion-selective vibrating microelectrodes (the MIFE technique, University of Tasmania, Hobart, Australia), essentially as described in our previous publications (Chen et al., 2007; Cuin et al., 2008). Barley seedlings were grown in an aerated Basic Salt Media (BSM) solution (0.5 mM KCl + 0.1 mM CaCl_2_, pH 5.6) in the dark for 3 days at room temperature (25 ± 1°C). At that stage, 1 mM MnCl_2_ was added to the BSM, and plants were grown for up to 3 more days in the presence of Mn. Roots of intact seedlings were mounted in a 10 mL perspex measuring chamber filling with the appropriate solution (BSM for control; BSM + 1 mM MnCl_2_ for Mn treatment) 1 h prior to measurement. Ion-selective microelectrodes were position 40 μm above the root surface, with their tips separated by ca. 2 μm. Ion fluxes were measured by a slow (5 s half-cycle) square-wave movement of electrodes between two positions, close to (40 μm) and away from (80 μm) the root surface. Net ion fluxes were measured from the mature root epidermis ~20 mm from the root tip. The potential difference between two positions was recorded by the MIFE CHART software and converted to the electrochemical potential difference using the calibrated Nernst slope of the electrodes. Net ion fluxes were calculated from the electrochemical potential difference using cylindrical diffusion geometry by the MIFEFLUX program. Net ion fluxes were measured for 10 min, and the steady-state fluxes were calculated by averaging the values over the last 5 min.

### Soil redox potential

Soil redox potential (or oxidation-reduction potential, ORP) was measured using an ORP electrode connected to a Handheld Multi-Parameter (LabNavigator, Forston Labs, Colorado, USA) before treatment (“drained control”), and after 3 and 14 days of waterlogging, both in the presence and absence of NaCl. To measure the ORP for drained controls, a 5 cm-deep hole was dug in the soil and the ORP electrode was carefully placed in the hole with the diaphragm touching the humid soil surface. All measurements were taken as close to roots as practically possible, without causing any damage. Each measurement was recorded for 180 s with one reading per second, and a reliable ORP value was achieved by averaging the readings from the last 120 s. These measurements were conducted about 1 h after watering.

### Fe and Mn concentrations in the soil solution

Soil solutions for determining Fe and Mn concentrations were sampled at the time of ORP measurement. For the WL and NaCl/WL treatments, soil solution was taken from five spots for each container, and combined into one collective sample for Fe and Mn determination. To determine Fe and Mn concentrations in the drained controls, a 1:2.5 (v/v) soil:solution mix was made (using tap water for clay, and ½ Hoagland nutrient solution for vermiculite), stirred for 30 min at room temperature, then decanted. All samples were then filtered, and Fe and Mn concentrations were determined using the Atomic Absorption Spectroscopy (AAS) technique (Avanta Σ, GBC Scientific Equipment, Braeside, Australia). Five replicates of collective samples were taken for each treatment.

### Statistical analysis

Statistical analysis was performed by the statistical package, IBM SPSS Statistics 20 (IBM, New York, USA). All data in the figures and tables are given as means ± SD. The Multivariate General Linear Model was used to confirm the significance of the factors (soil types, treatments, and varieties). Significant differences between treatments on the mean of the two varieties were compared using Duncan's multiple range tests.

## Results

### Plant growth

**Figure 1:**
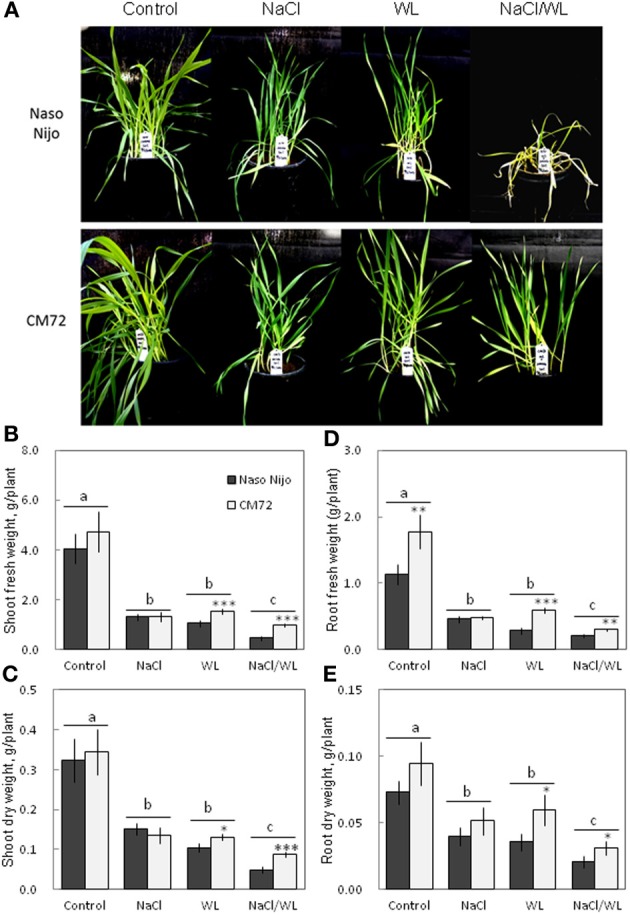
**Effects of separate and combined salinity and waterlogging stresses on growth of two contrasting barley cultivars in sandy loam**. Dark bars, Naso Nijo; light bars, CM72. One-week old barley seedlings were subjected to one of four treatments: Control (no NaCl; well drained), NaCl (well-drained pots watered with 200 mM NaCl solution), WL (submerged pots; no NaCl), NaCl/WL (pots submerged in 200 mM NaCl solution). Plants were harvested for morphological **(A)** and biomass analysis **(B–E)** 14 days after the onset of treatment. For growth condition and media composition, refer to the Materials and Methods section. Mean ± SD (*n* = 5). Different lower case letters indicate the significant difference between treatments (averaged for both genotypes) at *P* < 0.01. Asterisks indicate the significant difference between cultivars within treatment at ^*^ *P* < 0.01, ^**^P < 0.001, and ^***^P < 0.0001.

**Figure 2:**
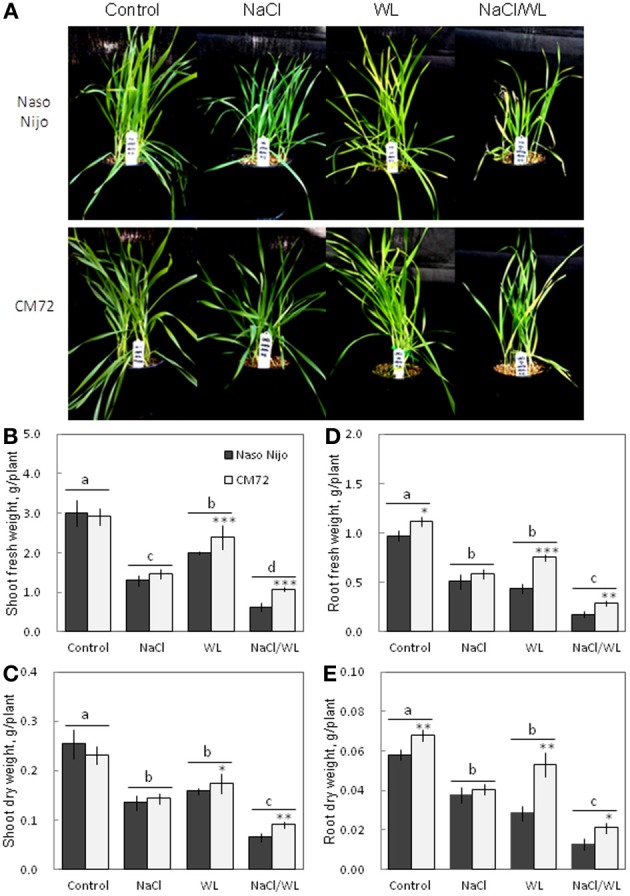
**Effects of separate and combined salinity and waterlogging stresses on growth of two contrasting barley cultivars in vermiculite**. Dark bars, Naso Nijo; light bars, CM72. One-week old barley seedlings were subjected to one of four treatments: Control (no NaCl; well drained), NaCl (well-drained pots watered with 200 mM NaCl solution), WL (submerged pots; no NaCl), NaCl/WL (pots submerged in 200 mM NaCl solution). Plants were harvested for morphological **(A)** and biomass analysis **(B–E)** 14 days after the onset of treatment. For growth conditions and media composition, refer to the Materials and Methods section. Mean ± SD (*n* = 5). Different lower case letters indicate the significant difference between treatments (averaged for both genotypes) at *P* < 0.01. Asterisks indicate the significant difference between cultivars within treatment at ^*^P < 0.01, ^**^P < 0.001, and ^***^P < 0.0001.

Plant growth was significantly reduced by all stress treatments (Figures 1, 2). The combined NaCl/WL treatment showed the most severe effect on plant growth. When grown in sandy loam, the average shoot FW and DW of the two varieties treated by NaCl/WL were reduced by 81 and 76% relative to the control, respectively, with the corresponding values for roots being 85 and 77% (Figures 1B–E). Plants grown in vermiculite performed better under combined NaCl/WL treatment, with a 71 and 32% reduction in the shoot FW and DW and 78 and 73% reduction in root FW and DW, respectively (Figures 2B–E). The genotypic difference in tolerance to the combined stress was clearly visible when plants were grown in sandy loam. After 14 days of NaCl/WL treatment, most Naso Nijo plants had died, and the plant FW was only 12% of that of control (Figures 1A,C). However, in the CM72 plants, only some moderately stressed symptoms were observed and 25% FW of the control was maintained.

### Chlorophyll content and fluorescence

**Figure 3:**
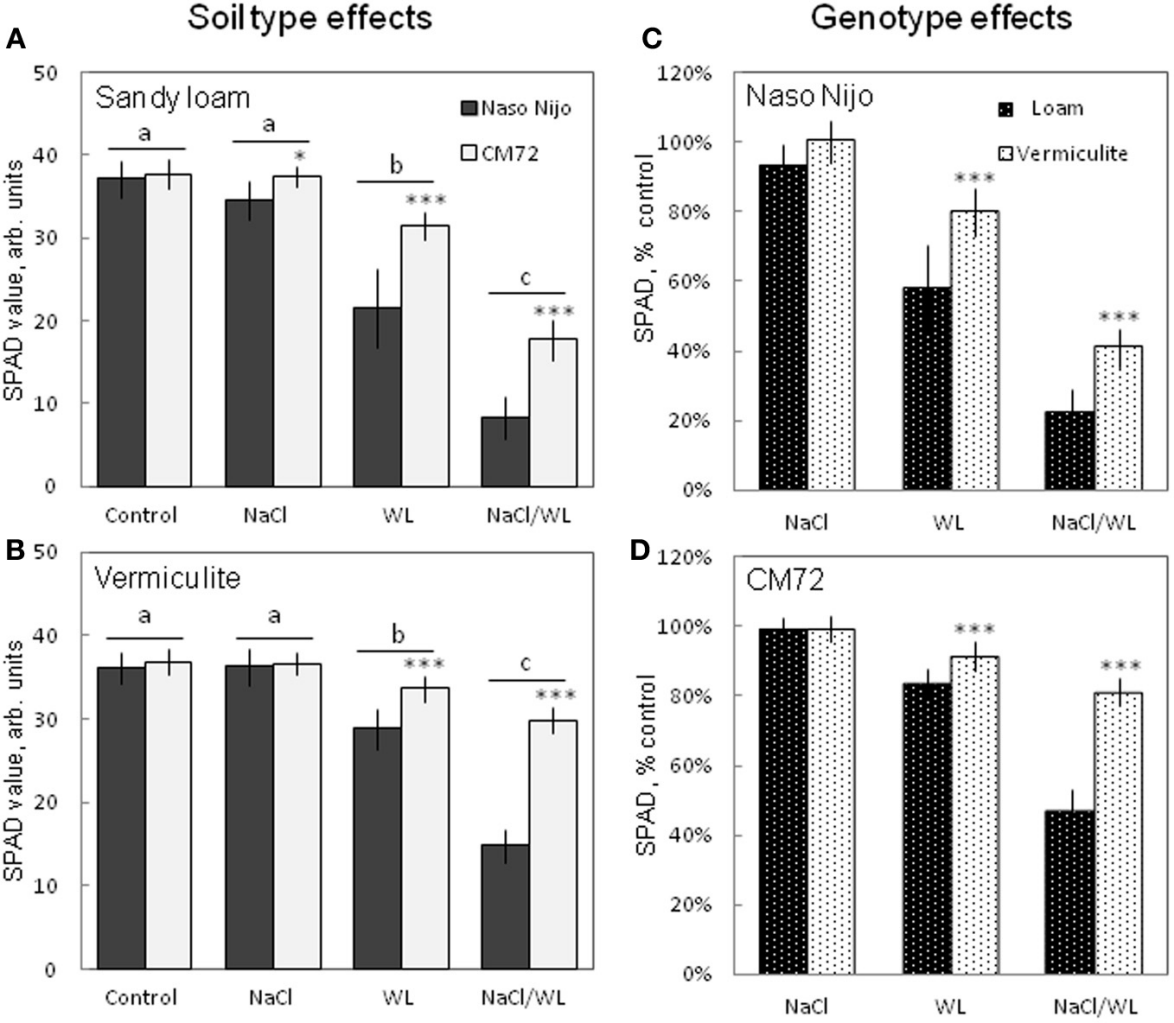
**Effects of separate and combined salinity and waterlogging stresses on chlorophyll content (SPAD values) of two contrasting barley cultivars**. Measurements were taken 14 days after the onset of treatment. **(A,B)** Soil type effects. Dark bars, Naso Nijo; light bars, CM72. **(C,D)** Genotype effects. Dark bars, sandy loam; light bars, vermiculite. For growth conditions, details of treatments, and media composition, refer to the Materials and Methods section. Mean ± SD (*n* = 12). Different lower case letters indicate the significant difference between treatments (averaged for both genotypes) at *P* < 0.01. Asterisks indicate the significant difference between cultivars within the treatment at ^*^P < 0.01 and ^***^P < 0.0001.

After 2 weeks of treatment, NaCl alone had no significant (at *P* < 0.01) effect on the leaf chlorophyll content (SPAD value) for either variety grown in either growth media. However, WL and NaCl/WL treatments caused a massive reduction in the chlorophyll content (Figure 3) in both varieties. Sandy loam-grown plants were affected more than plants grown in vermiculite (ca. 40% more reduction in NaCl/WL treatment, Figures 3C,D). The combined NaCl/WL treatment was more detrimental compared with WL alone in both varieties (Figures 3A,B). The observed decline in chlorophyll content was genotype-specific, with chlorophyll content in CM72 plants being significantly higher than that in Naso Nijo plants for each treatment, except for the control (Figures 3A,B).

**Figure 4:**
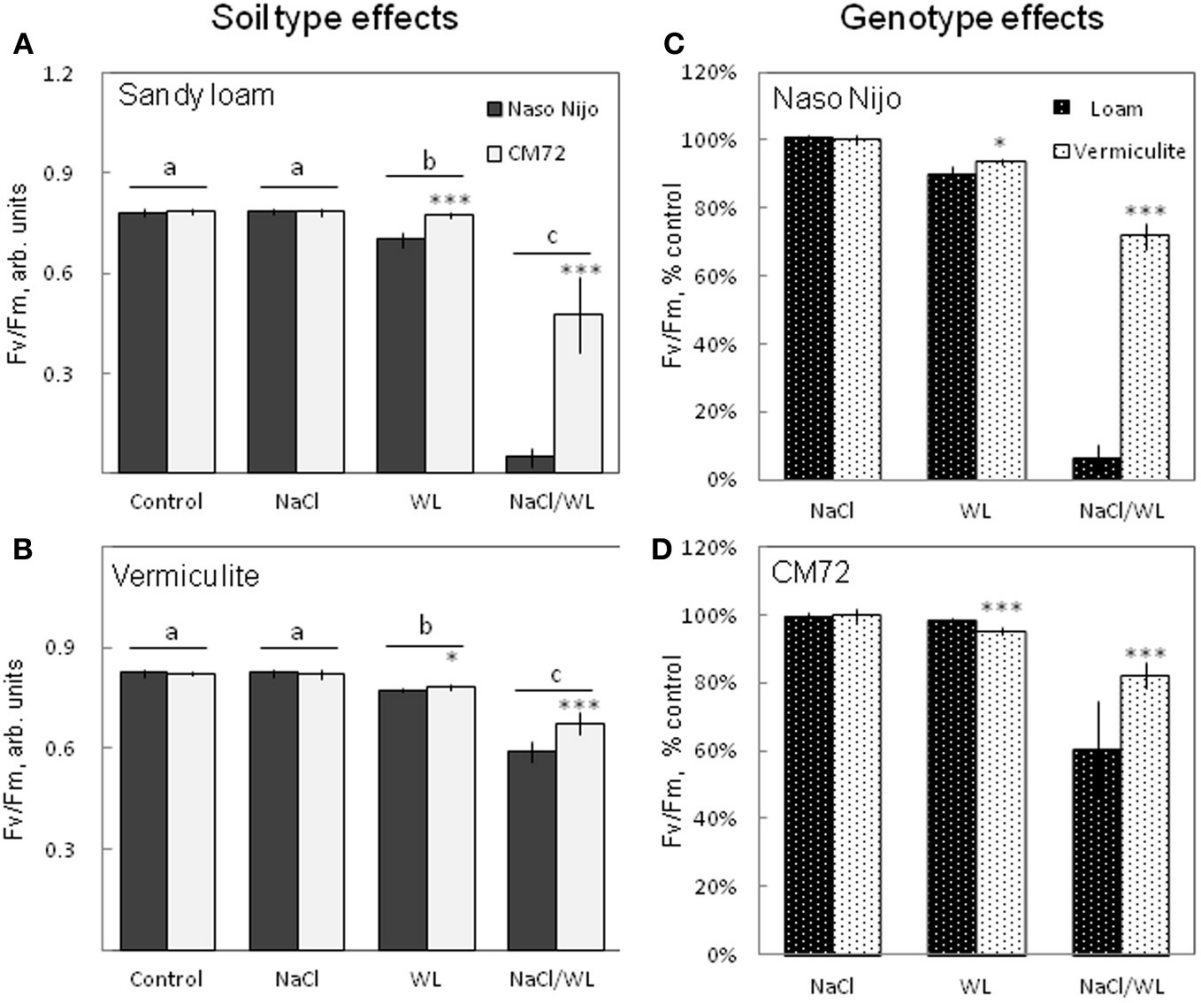
**Effects of separate and combined salinity and waterlogging stresses on maximum photochemical efficiency of PSII (Fv/Fm chlorophyll fluorescence values) of two contrasting barley cultivars**. Measurements were taken 14 days after the onset of treatment. **(A,B)** Soil type effects. Dark bars, Naso Nijo; light bars, CM72. **(C,D)** Genotype effects. Dark bars, sandy loam; light bars, vermiculite. For growth conditions, details of treatments, and media composition, refer to the Materials and Methods section. Mean ± SD (*n* = 12). Different lower case letters indicate the significant difference between treatments (averaged for both genotypes) at *P* < 0.01. Asterisks indicate the significant difference between cultivars within the treatment at ^*^P < 0.01 and ^***^P < 0.0001.

The maximum photochemical efficiency of PSII (chlorophyll fluorescence *F*v/*F*m value) was also significantly affected by WL and NaCl/WL treatments (Figure 4). A substantial decline in *F*v/*F*m value was reported for both treatments (Figures 4A,B). None of these parameters, however, was affected by NaCl alone (at *P* < 0.01, Figures 4A,B). On averaging the two varieties, the effect of the combined NaCl/WL stress was much more severe than WL alone (at *P* < 0.01, Figures 4A,B). CM72 plants were less sensitive to both WL and combined NaCl/WL stresses than Naso Nijo (Figures 4A,B). Stress effects were strongly influenced by the growth conditions (sandy loam vs. vermiculite), with a much stronger decline in *F*v/*F*m observed in sandy loam-grown plants (66% more reduction for Naso Nijo and 22% more for CM72 under sandy loam conditions than those grown in vermiculite, Figures 4C,D).

### Leaf chlorosis and necrosis

**Figure 5:**
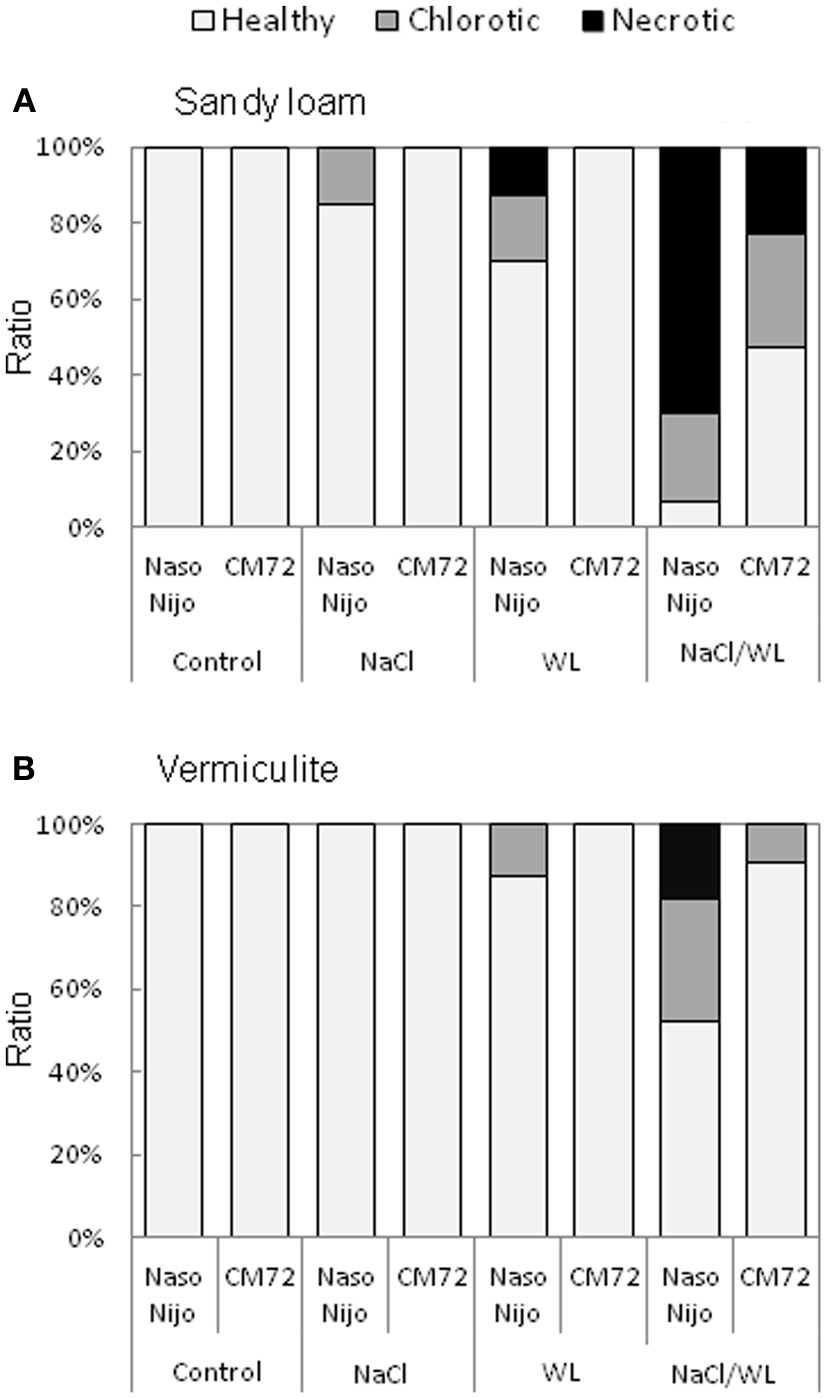
**Effects of separate and combined salinity and waterlogging stresses on the ratio of chlorotic and necrotic leaves of two contrasting barley cultivars**. Ratio of chlorotic and necrotic leaves were calculated according to the numbers of total, chlorotic and necrotic leaves on plants grown in sandy loam **(A)** and vermiculite **(B)** 14 days after the onset of treatment (dark bars, Naso Nijo; light bars, CM72). For growth conditions, details of treatments, and media composition, refer to the Materials and Methods section. Mean ± SD (*n* = 12).

Leaf chlorosis and necrosis are important visible symptoms associated with abiotic stresses. As shown in Figure 5, leaf chlorosis and necrosis were induced by WL treatment and exacerbated by the combined NaCl/WL stress. Variety CM72 performed much better compared with Naso Nijo. As such, no chlorotic or necrotic leaves were present in CM72 plants under WL treatment, while a substantial percentage of Naso Nijo leaves were affected by WL (Figure 5A). Under combined NaCl/WL stress, half the CM72 leaves showed visual stress symptoms, while in the more sensitive Naso Nijo, over 90% leaves were either chlorotic or nectrotic (Figure 5A). Visual stress symptoms were more pronounced in the sandy loam-grown plants (Figure 5A) compared with those grown in vermiculate (Figure 5B). Taking the combined NaCl/WL treatment as an example, Naso Nijo had over 70% necrotic leaves when grown in sandy loam but only 18% in vermiculite; for CM72 the corresponding values were 22 and 0% (Figure 5).

### Leaf water content and sap osmolality

**Figure 6:**
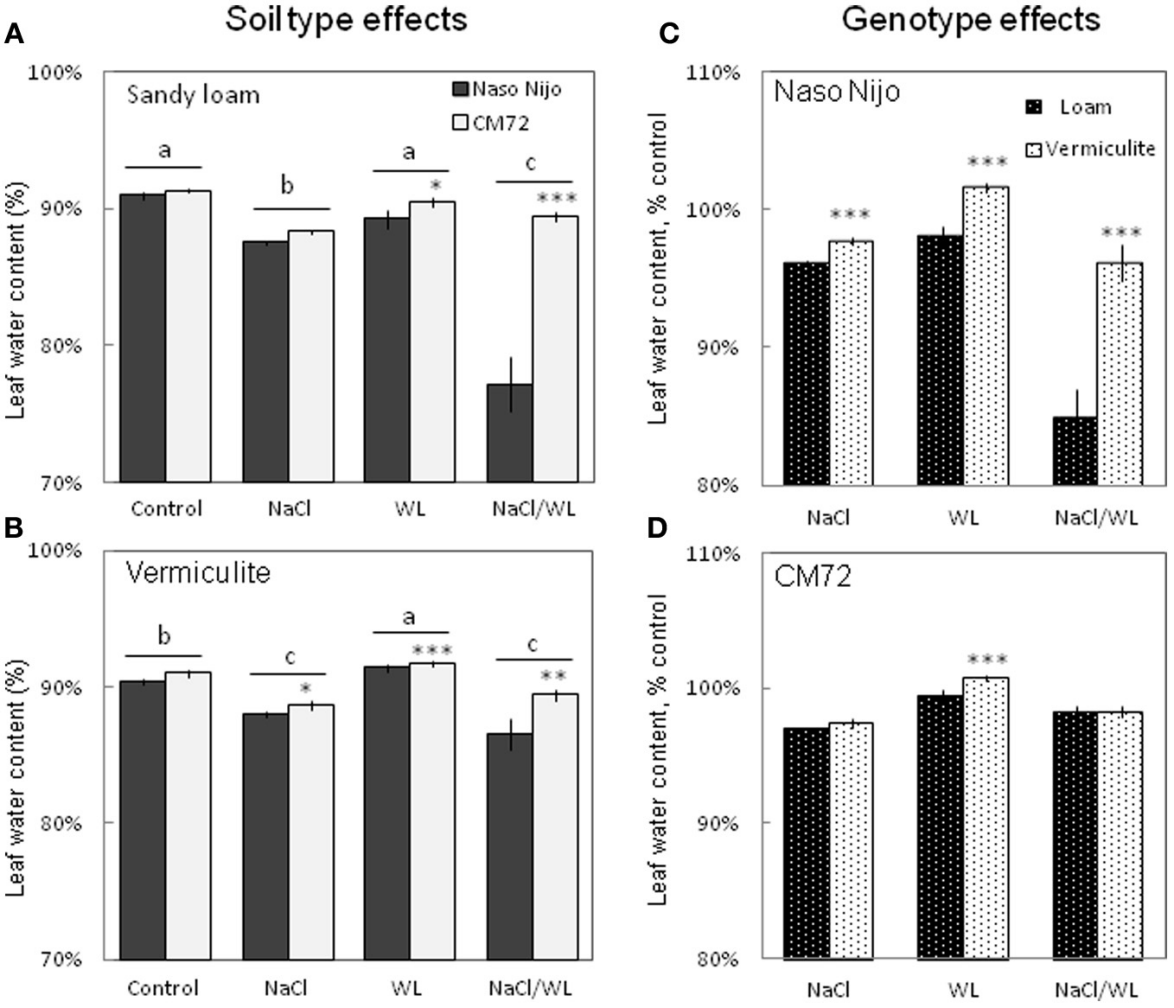
**Effects of separate and combined salinity and waterlogging stresses on leaf water content of two contrasting barley cultivars. (A,B)** Soil type effects. Dark bars, Naso Nijo; light bars, CM72. **(C,D)** Genotype effects. Dark bars, sandy loam; light bars, vermiculite. For growth conditions, details of treatments, and media composition, refer to the Materials and Methods section. Mean ± SD (*n* = 5). Different lower case letters indicate the significant difference between treatments (averaged for both genotypes) at *P* < 0.01. Asterisks indicate the significant difference between cultivars within the treatment at ^*^P < 0.01, ^**^P < 0.001, and ^***^P < 0.0001.

Leaf WC was significantly decreased by NaCl and NaCl/WL treatments under both growth conditions (Figure 6). At the same time, WL treatment did not significantly affect leaf WC under sandy loam conditions (at *P* < 0.01, Figure 6A), and even slightly increased leaf WC in plants grown in vermiculite (Figure 6B). A significant (at *P* < 0.01) difference in leaf WC between the contrasting varieties was observed for each treatment except the control in this experiment (Figures 6A,B). The growth conditions (sandy loam vs. vermiculite) showed a significant impact on leaf WC, but only in the sensitive variety Naso Nijo (Figures 6C,D).

**Figure 7:**
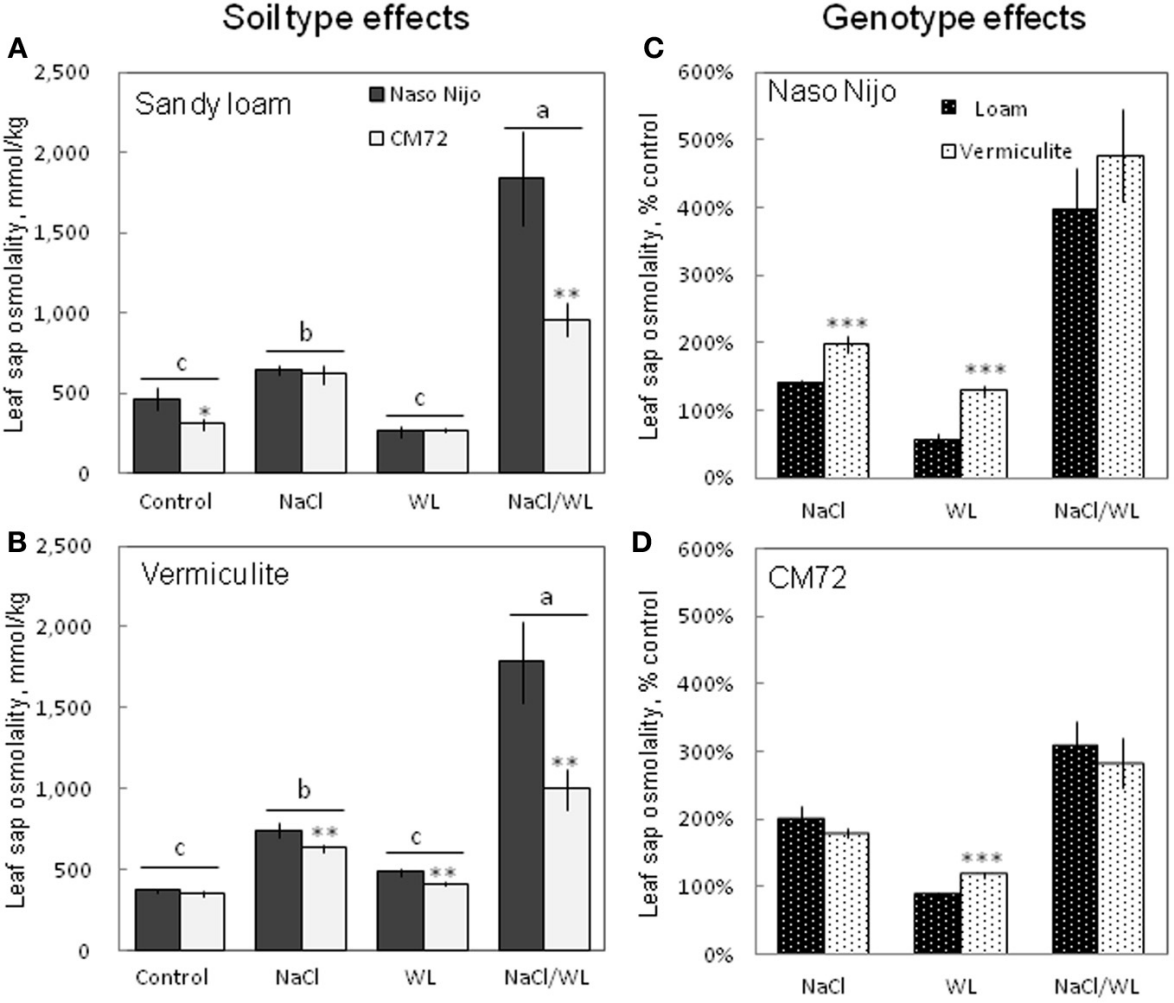
**Effects of separate and combined salinity and waterlogging stresses on leaf sap osmolality of two contrasting barley cultivars. (A,B)** Soil type effects. Dark bars, Naso Nijo; light bars, CM72. **(C,D)** Genotype effects. Dark bars, sandy loam; light bars, vermiculite. For growth conditions, details of treatments, and media composition, refer to the Materials and Methods section. Mean ± SD (*n* = 5). Different lower case letters indicate the significant difference between treatments (averaged for both genotypes) at *P* < 0.01. Asterisks indicate the significant difference between cultivars within the treatment at ^*^P < 0.01, ^**^P < 0.001 and ^***^P < 0.0001.

To a large extent, changes in the leaf WC were mirrored by changes in leaf sap osmolality (Figure 7). Much higher leaf sap osmolality (significant at *P* < 0.01) was measured in plants grown in the presence of NaCl (i.e., in both NaCl and NaCl/WL treatments), while no significant change in leaf osmolality was found for WL treatment as compared to the control. The combination of waterlogging and salt stress not only induced a massive increase in leaf sap osmolality, but also allowed a clear differentiation between the contrasting varieties, with about a 2-fold difference (significant at *P* < 0.001) between them (Figures 7A,B). No significant (at *P* < 0.01) effect of growth media (sandy loam vs. vermiculate) on leaf sap osmolality was found (Figures 7C,D).

### Sap Na^+^ and K^+^ contents

**Figure 8:**
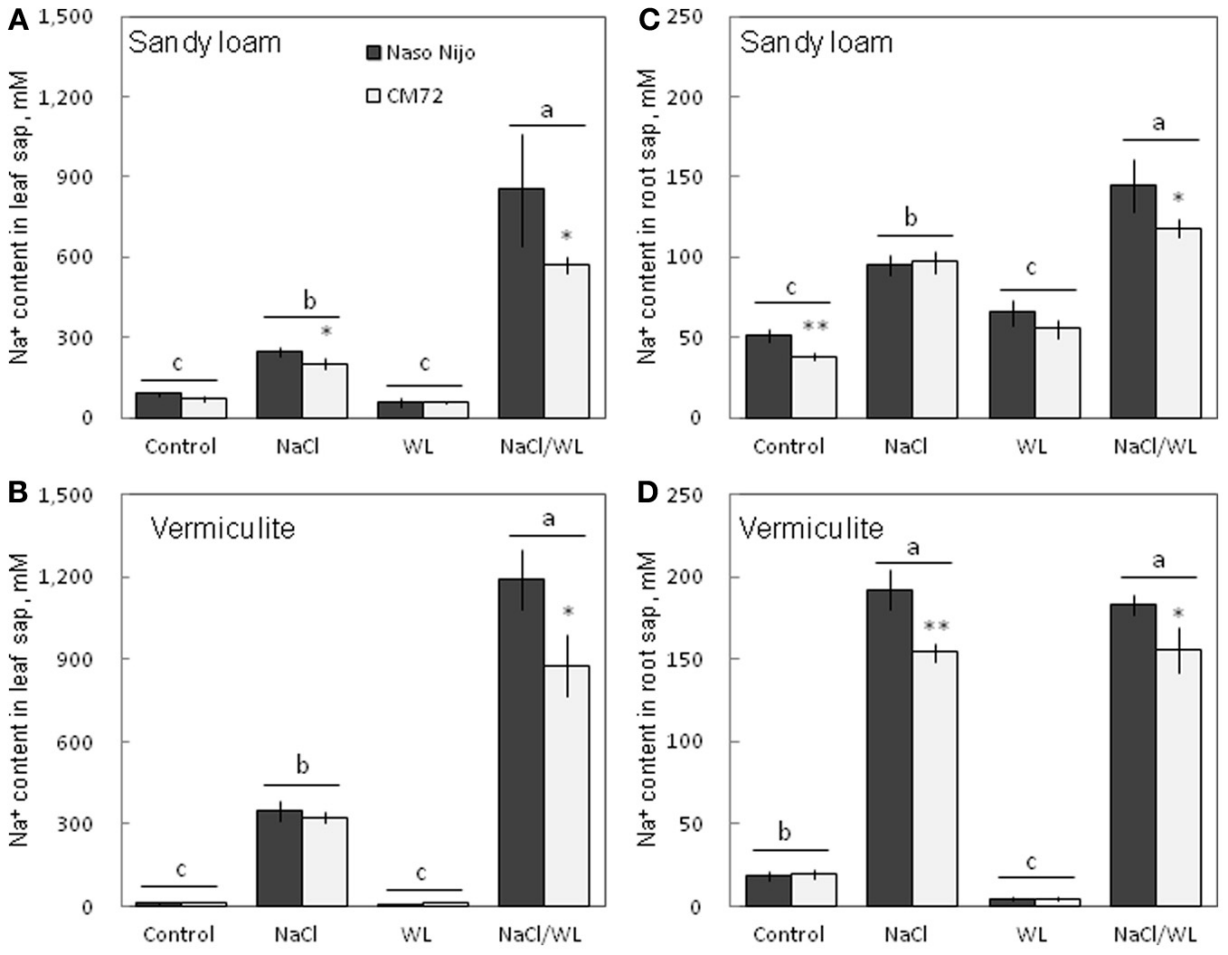
**Effects of separate and combined salinity and waterlogging stresses on tissue Na^+^ content of two contrasting barley cultivars**. **(A,B)** Soil type effects. Dark bars, Naso Nijo; light bars, CM72. **(C,D)** Genotype effects. Dark bars, sandy loam; light bars, vermiculite. For growth conditions, details of treatments, and media composition, refer to the Materials and Methods section. Mean ± SD (*n* = 5). Different lower case letters indicate the significant difference between treatments (averaged for both genotypes) at *P* < 0.01. Asterisks indicate the significant difference between cultivars within treatment at ^*^P < 0.01, ^**^P < 0.001.

**Figure 9:**
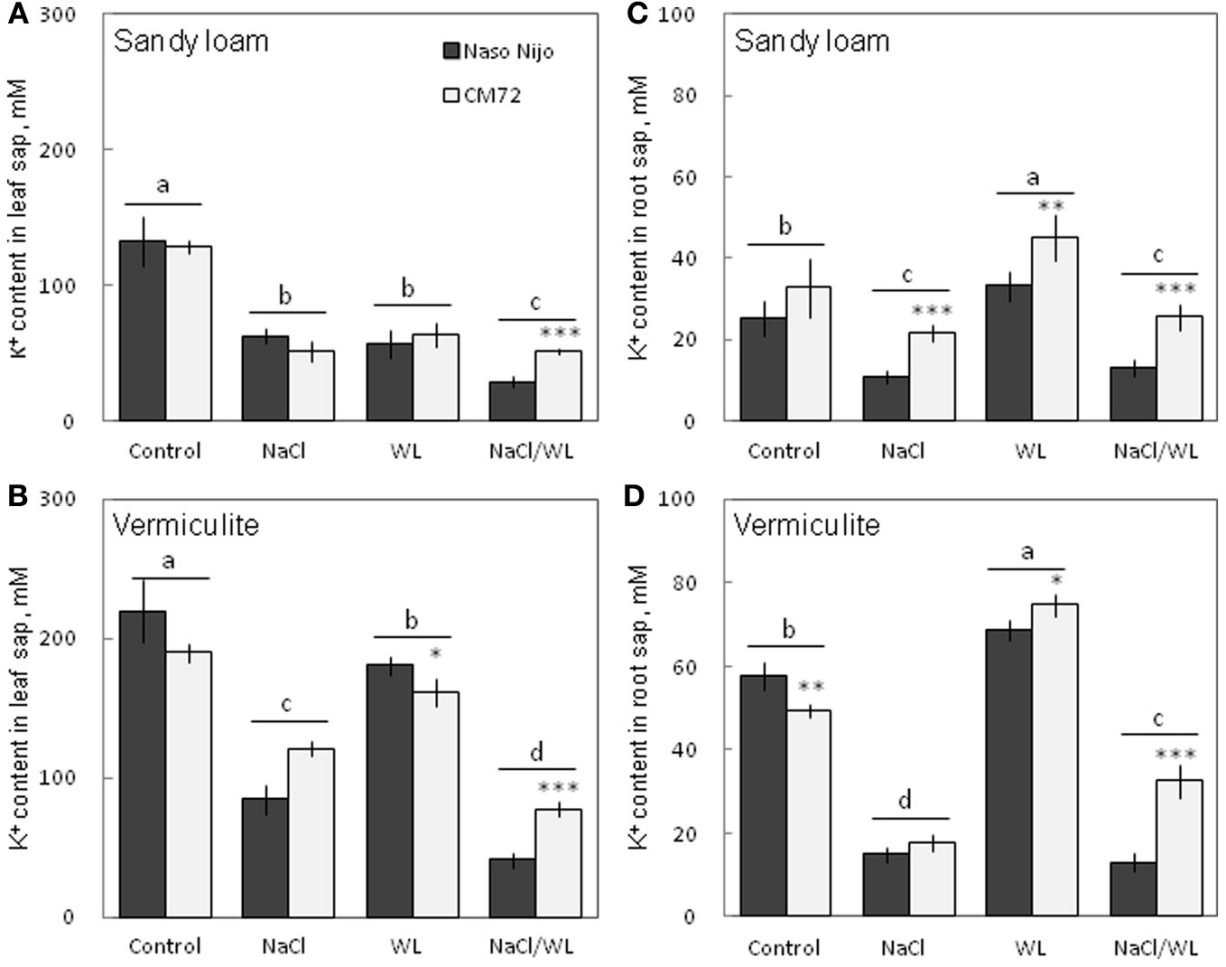
**Effects of separate and combined salinity and waterlogging stresses on tissue K^+^ content of two contrasting barley cultivars. (A,B)** Soil type effects. Dark bars, Naso Nijo; light bars, CM72. **(C,D)** Genotype effects. Dark bars, sandy loam; light bars, vermiculite. For growth conditions, details of treatments, and media composition, refer to the Materials and Methods section. Mean ± SD (*n* = 5). Different lower case letters indicate the significant difference between treatments (averaged for both genotypes) at *P* < 0.01. Asterisks indicate the significant difference between cultivars within treatment at ^*^P < 0.01, ^**^P < 0.001, and ^***^P < 0.0001.

As expected, NaCl treatment caused dramatic increases in the Na^+^ content (Figure 8) but a decrease in the K^+^ content (Figure 9) for both the leaf and root (at *P* < 0.01). Compared with NaCl alone, the combined NaCl/WL treatment further increased the leaf Na^+^ content (about a 1.7–2-fold increase on average of the two varieties, Figures 8A,B), but decreased leaf K^+^ content (about 27 to 44% decrease on average of the two varieties, Figures 9A,B). However, the combined NaCl/WL treatment did not cause further changes to root Na^+^ (Figures 8C,D) and K^+^ (Figures 9C,D) contents. WL treatment induced a significant decrease in leaf K^+^ content and a significant increase in root K^+^ content (at *P* < 0.01, Figure 9) as compared to control, while no significant change was found in Na^+^ content (at *P* < 0.01, Figure 8). A clear differentiation in Na^+^ and K^+^ contents between the contrasting varieties appeared under the combined NaCl/WL treatment. Relative to Naso Nijo, the tolerant variety CM72 had a 27–33% lower Na^+^ content (at *P* < 0.01, Figures 8A,B) and 77–88% higher K^+^ content (at *P* < 0.0001, Figures 9A,B) in leaves. For roots, the corresponding values were 15–18% (at *P* < 0.0001, Figures 8C,D) and 94–150% (at *P* < 0.0001, Figures 9C,D). The growth conditions (sandy loam vs. vermiculite) showed a significant impact on both Na^+^ and K^+^ contents. The vermiculite-grown plants contained much more Na^+^ and K^+^ in both the leaves and roots compared to those grown in sandy loam under saline conditions (both NaCl and NaCl/WL treatments) (Figures 8, 9). Taking CM72 in the combined NaCl/WL treatment as an example, plants grown in vermiculite had a 54% higher leaf Na^+^ and 32% higher root Na^+^ contents than in sandy loam (Figure 8). For K^+^, the corresponding values were 50 and 27% (Figure 9).

### Root ATP content

**Figure 10:**
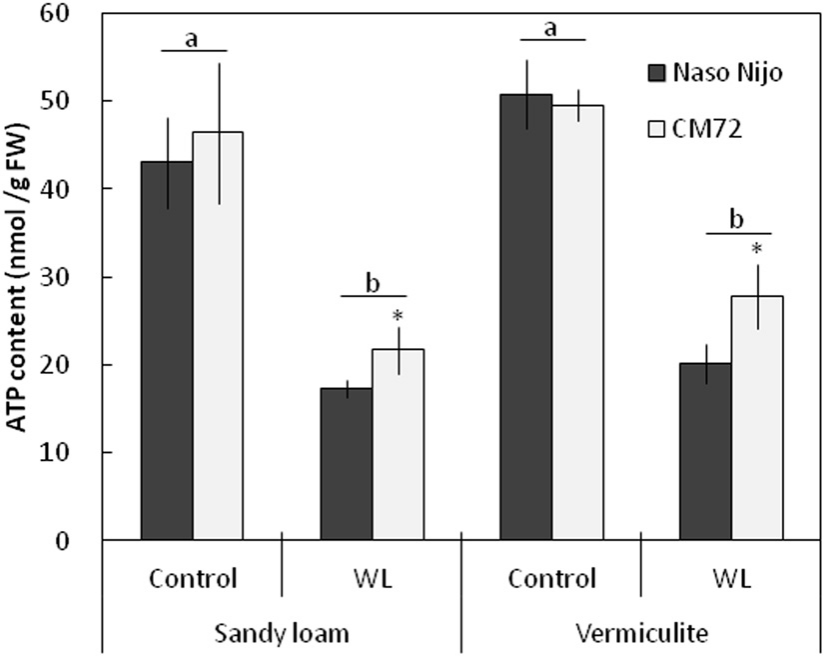
**ATP content in barley roots grown under normoxic and hypoxic conditions for 14 days**. Closed bars—Naso Nijo; open bars—CM72. Mean ± SD (*n* = 4 plants). Different lower case letters indicate the significant difference between treatments (averaged for both genotypes) at *P* < 0.01. Asterisk indicate the significant difference between cultivars within the treatment at ^*^P < 0.05.

Under normoxic conditions, the ATP content in plant roots was not significantly (at *P* < 0.05) different between genotypes (Figure 10). ATP content was also not significantly (at *P* < 0.05) different between two soil types, ranging from 42 to 51 nmol ATP/g FW (Figure 10). Two weeks of hypoxia resulted in a 2–3-fold decrease in the root ATP content. No significant (*P* < 0.05) effect of soil type on ATP decline was observed (Figure 10). Hypoxia-exposed CM72 plants were capable of maintaining a slightly higher (significant at *P* < 0.05) root ATP level compared with Naso Nijo variety.

### Changes in soil chemistry

**Figure 11:**
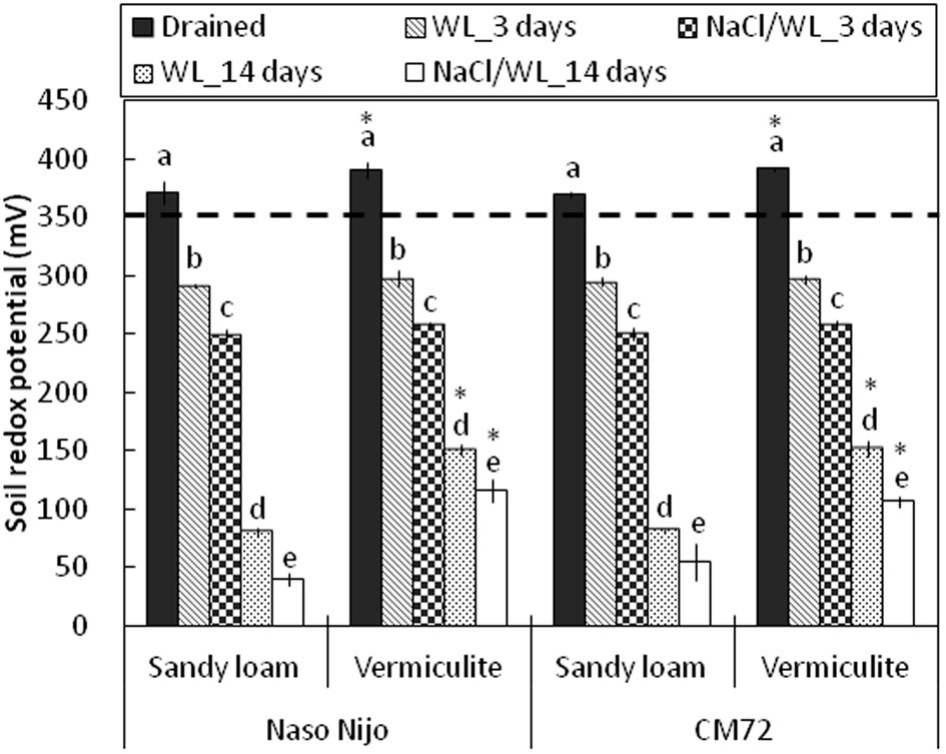
**Soil redox potential during waterlogging (submerged; no NaCl) and combined salinity and waterlogging (submerged in 200 mM NaCl solution) treatments**. Soil redox potential measurements were taken before treatment (drained), and 3 days and 14 days after commencing the treatments. Mean ± SD (*n* = 5). Different lower case letters indicate the significant difference between treatments within soil type at *P* < 0.01. Asterisk indicates the significant differences between sandy loam and vermiculite within variety a *P* < 0.01. Horizontal dashed line indicates the threshold level for anaerobic condition (below 350 mV).

Development of hypoxia was accompanied by a progressive decline in the soil redox potential (ORP), from 391 ± 1.5 mV to less than 50 mV, depending on the soil type and duration of waterlogging (Figure 11). No significant (at *P* < 0.05) differences were found for the ORP values between two soil types after 3 days of WL stress. When pots were submerged for 2 weeks however, ORP values were significantly (*P* < 0.01) lower in sandy loam compared with vermiculite (80 ± 4.4 mV vs. 151 ± 4.5 mV, respectively). The presence of salt further reduced the appropriate ORP values for each treatment by 30–40 mV, but the general trends remained the same.

**Figure 12:**
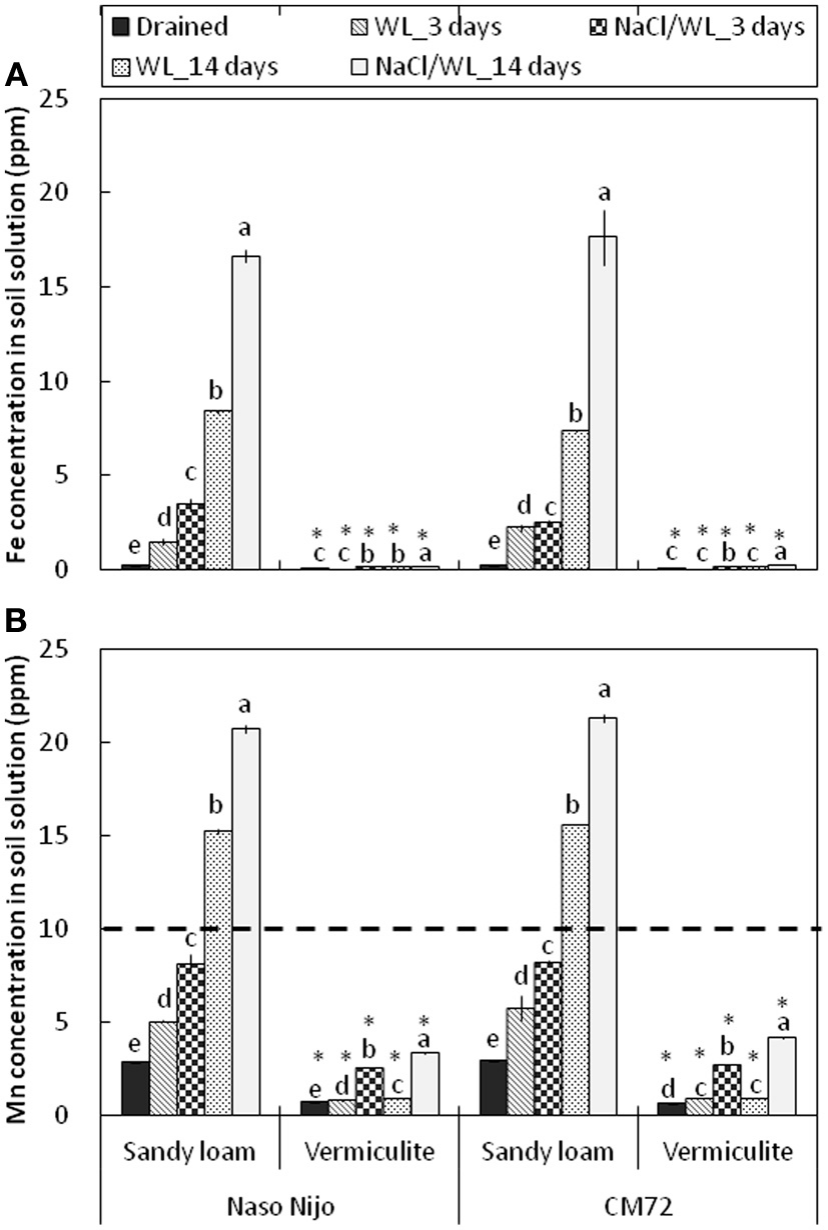
**Concentrations of Fe (A) and Mn (B) in the soil solution in pots subjected to waterlogging (submerged; no NaCl) and combined salinity and waterlogging (submerged in 200 mM NaCl solution)**. Soil samples were taken on days 3 and 14 since commencement of treatment and compared with those taken from control treatment. Mean ± SD (*n* = 5). Different lower case letters indicate the significant difference within the soil type at *P* < 0.01. Asterisk indicates the significant differences between sandy loam and vermiculite within variety at *P* < 0.01.

Consistent with the decline in the ORP values, waterlogging induced a very substantial increase in both Mn and Fe content in the sandy loam soil (Figure 12). After 2 weeks of waterlogging, the Mn content rose from 2.8 ± 0.01 to 15.4 ± 0.06 ppm in waterlogging treatment alone, and to 21.8 ± 0.08 ppm in combined NaCl/WL treatment, exceeding the threshold level considered to be toxic for cereals (10 ppm; Setter et al., 2009; shown as a dotted line). Also dramatic was the increase in solution Fe content (from 0.23 ± 0.02 ppm to 16.8 ± 0.05 ppm). No physiologically relevant increase in either Mn or Fe content was found in waterlogged vermiculite (Figure 12). The presence of NaCl in the media consistently increased the Mn and Fe content in the soil solution by around 2-fold for each treatment.

### Root ion flux changes

**Figure 13:**
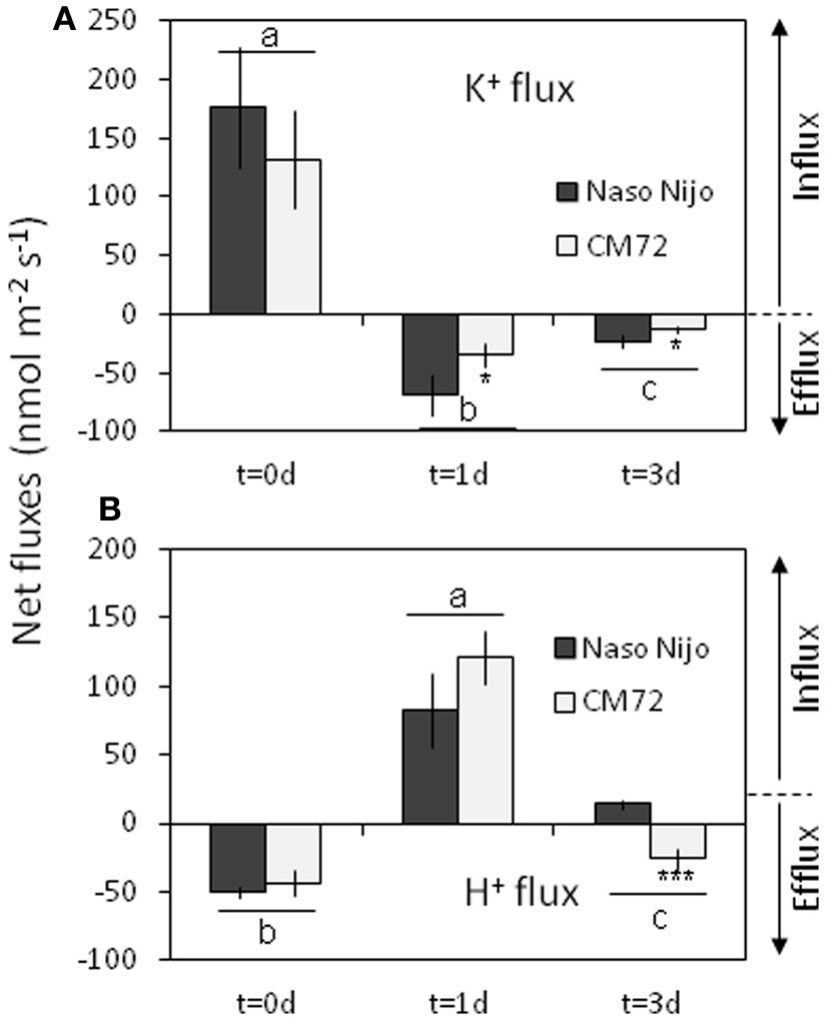
**Net steady-state K^+^ (A) and H^+^ (B) fluxes measured from mature barley root epidermis in control (day 0) and after one and three days exposure to 1 mM Mn**. Mean ± SD (*n* = 6). Different lower case letters indicate the significant difference between treatments (averaged for both genotypes) at *P* < 0.05. Asterisks indicate the significant difference between cultivars within treatment at ^*^P < 0.05 and ^***^P < 0.001.

A strong net K^+^ uptake of 130–180 nmol m^−2^s^−1^ (depending on gentotype) was measured from the barley root epidermis in the control (Figure 13A). After 1 days of root treatment with 1 mM Mn, K^+^ fluxes turned to a net efflux of 35–70 m^−2^s^−1^ and remained negative (net K^+^ loss) for at least a few more days (Figure 13A). This Mn-induced K^+^ efflux was 2-fold higher in the waterlogging-sensitive Naso Nojo variety (significant at *P* < 0.05). Changes in H^+^ flux mirrored the changes in the K^+^ flux. A strong net H^+^ efflux of around 50 nmol m^−2^s^−1^ was measured from the control roots (Figure 13B). This efflux was sensitive to 1 mM vanadate, a known blocker of H^+^-ATPase (data not shown). Mn treatment reversed the H^+^ efflux into a net H^+^ influx after 1 day (Figure 13B). At day 3 of Mn treatment, the CM72 genotype was capable of restoring its net H^+^ extrusion (albeit at a reduced rate), while the WL-sensitive variety Naso Nijo still displayed a net H^+^ influx (Figure 13B).

## Discussion

### Disturbance to K^+^/Na^+^ homeostasis is central for the severe effects of combined salinity and waterlogging stress

In this study, a much more dramatic increase in Na^+^ and decrease in K^+^ content were observed in both roots and leaves of barley plants grown under a combined salinity and waterlogging stress compared to plants grown with salinity under drained condition (Figures 8, 9). Intracellular K^+^/Na^+^ homeostasis is often named as a key determinant of plant salinity tolerance (Maathuis and Amtmann, 1999; Shabala and Cuin, 2008). The key players in this process are the plasma membrane SOS1 Na^+^/H^+^ antiporters that actively expel Na^+^ from the cytosol (Shi et al., 2002) and depolarization-activated outward-rectifying (GORK in Arabidopsis; Ache et al., 2000) channels, responsible for K^+^ retention in the cytosol (Shabala and Cuin, 2008). The operation of both of these transporters is critically dependent on oxygen availability. Indeed, SOS1 activity relies on existence of steep H^+^ gradients across the plasma membrane, fueled by the plasma membrane H^+^-ATPase activity (Palmgren and Harper, 1999). The H^+^-pump is also the main electrogenic factor essential for maintaining a highly negative membrane potential value, thus keeping GORK channels closed. Salinity by itself causes a substantial membrane depolarization (by 60–80 mV; Shabala and Cuin, 2008). As the oxygen is gradually used up (under waterlogged conditions), root O_2_ deficiency would restrict aerobic respiration, so the production of ATP would be dramatically reduced (from 30 to 36 mol ATP via mitochondrial oxidative phosphorylation to 2–4 mol ATP via glycolysis per hexose, Bailey-Serres and Voesenek, 2008). Indeed, the ATP content dropped 2–3-fold in waterlogging-affected roots (Figure 10). This reduction will compromise a plant's ability to fuel H^+^-ATPases, with major implications to both Na^+^ exclusion and K^+^ retention, as discussed above. Overall, unfavorable Na^+^/K^+^ ratios in plant will affect plant metabolism, resulting in a significant decrease in chlorophyll content (Figure 3) and photochemical efficiency of PSII (Figure 4); drastic increases in the number of chlorotic and necrotic leaves (Figure 5) and leaf sap osmolality (Figure 7); and ultimately reduced growth of roots and shoots (Figures 1, 2).

### Soil type affects the adverse effects of combined waterlogging and salinity stress

As the soil redox potential is reduced under waterlogged conditions, manganese dioxide (Mn^4+^) and the insoluble ferric (Fe^3+^) hydroxides are reduced to the soluble manganous (Mn^2+^) and ferrous (Fe^2+^) ions (Khabaz-Saberi et al., 2006; Setter et al., 2009; Hernandez-Soriano et al., 2012). In our case, a substantial increase in both Mn and Fe content in the soil solution was observed for the sandy loam, but not the vermiculite soil (Figure 12). Despite its importance as an essential micronutrient, excess Mn is damaging to the photosynthetic apparatus, interferes with uptake of other nutrients and may cause oxidative stress, resulting in chlorosis and necrosis in leaves and subsequently death of whole plants (Millaleo et al., 2010). Visual symptoms of Mn toxicity such as chlorosis and necrosis were observed in barley leaves of hydroponically-grown plants at concentrations as low as 50 μM (i.e., <5 ppm; Führs et al., 2010). In field experiments, the toxic threshold for cereals grown in drained soils was reported to be around 10 ppm of Mn (Setter et al., 2009). In our case, the Mn content in the sandy loam soil solution exceeded the latter threshold by day 14 for WL alone treatment, and was close to it around day 3 in the combined NaCl/WL treatment (Figure 12). Mn treatment also had major implications for K^+^ retention in barley roots within 24 h of exposure (Figure 13). Consequently, the dramatic increase in Mn concentration that we found in sandy loam (up to 20 ppm, Figure 12) under waterlogged conditions would likely induce a high risk of Mn toxicity to barley plants. Interestingly, the presence of NaCl in the soil exacerbated the effect of waterlogging on Mn availability, in a full agreement with the ORP data (Figure 11). The Fe content in the soil solution also increased steadily, approaching toxic levels. Together, when delivered to the shoot by the transpiration flow, these two toxic micronutrients could lead to increased ROS formation in green tissues (Millaleo et al., 2010; Keunen et al., 2011), impairing the photosynthetic machinery (Figure 4), reducing chlorophyll content (Figure 3) and enhancing the senescence process (Figure 5). Such elemental toxicity may be an additional (to unfavorable Na^+^/K^+^ balance in leaf tissues) factor contributing to the poor plant performance under combined WL/NaCl conditions in sandy loam. Elemental toxicity, however, is not a factor for vermiculate-grown plants, explaining their much better performance (compared with loam-grown plants) under combined stress conditions (Figures 1, 2).

So far, most studies dealing with the combined effects of salinity and waterlogging stress have been conducted with deoxygenated solution (stagnant agar solution or N_2_ aerated solution) or waterlogged sand (Barrett-Lennard, 2003; Teakle et al., 2006, 2010; Rogers et al., 2011). Being very useful to clarify the underlying mechanisms of this interaction, these results may be at some discrepancy with the real situation in the natural stressed environment, due to the lack of the factor of elemental toxicity, typically present in waterlogged soils. Therefore, for accurately evaluating and efficiently improving the tolerance of germplasm to combined salinity and waterlogging stress, it is necessary to carry out investigations and screening with the soil from the target environment rather than with artificial growing methods.

### Genotypic differences in responding to combined waterlogging and salinity stress and their potential utilization in saltland

It is estimated that over 370,000 barley germplasms are preserved as *ex situ* collections in worldwide representative genebanks (Saisho and Takeda, 2011). The genetic diversity of either salinity or waterlogging tolerance has been well proved amongst cultivated and even wild barley germplasms (Wu et al., 2011). However, little information about the genetic variation in a combined waterlogging and salinity stress has been accumulated. In the present study, a significant genetic difference in response to combined waterlogging and salinity stress was observed between CM72 and Naso Nijo. Regardless of the soil type, CM72 showed a much higher tolerance to combined WL/NaCl stress than Naso Nijo, as reflected by the lower reduction in root and shoot growth (Figures 1, 2), higher chlorophyll content (Figure 3) and fluorescence (Figure 4), fewer chlorotic and necrotic leaves (Figure 5), higher leaf WC (Figure 6), lower Na^+^, and higher K^+^ content in root and leaf sap (Figures 8, 9). The vast collection of barley germplasms and the recent completion of the barley genome sequencing (The International Barley Genome Sequencing Consortium, 2012) makes it possible to thoroughly explore the potential mechanisms of tolerance of different barley cultivars to individual or combined stress conditions and utilize the more tolerant lines to breed suitable varieties for the corresponding environment. Up to now, saltland agriculture has relied heavily on the use of pasture halophylic species such as *Atriplex, Maireana, Puccinellia, Thinopyrum, Lotus*, and *Melilotus* (Teakle et al., 2006; Bennett et al., 2009; Rogers et al., 2011). Barley is a crop that can be used not only for animal feed, but also for alcoholic and non-alcoholic beverages and human food. It is already known for its tolerance to stresses such as cold, drought, and salinity (Saisho and Takeda, 2011). It can be predicted that barley will be a potential food source in future for the increasing world population under deteriorating environments. To achieve this goal, truly tolerant varieties capable of performing without, or with only little yield reduction, under hostile soil conditions, such as combined salinity and waterlogging, should be selected or created by breeders.

#### Conflict of interest statement

The authors declare that the research was conducted in the absence of any commercial or financial relationships that could be construed as a potential conflict of interest.
